# Reduced bronchoalveolar macrophage phagocytosis and cytotoxic effects after controlled short-term exposure to wood smoke in healthy humans

**DOI:** 10.1186/s12989-023-00541-x

**Published:** 2023-07-31

**Authors:** Alva Hansson, Gregory Rankin, Oskari Uski, Maria Friberg, Jamshid Pourazar, Robert Lindgren, Natxo García-López, Christoffer Boman, Thomas Sandström, Annelie Behndig, Ala Muala

**Affiliations:** 1 Department of Public Health and Clinical Medicine, Umeå University, Umeå, Sweden; 2 Thermochemical Energy Conversion Laboratory, Department of Applied Physics and Electronics, Umeå University, Umeå, Sweden

**Keywords:** Air pollution, Biomass combustion, Wood smoke, Controlled human exposure, Bronchoscopy, Cytotoxicity, Macrophages, Phagocytosis, In vitro

## Abstract

**Background:**

Exposure to wood smoke has been shown to contribute to adverse respiratory health effects including airway infections, but the underlying mechanisms are unclear. A preceding study failed to confirm any acute inflammation or cell influx in bronchial wash (BW) or bronchoalveolar lavage (BAL) 24 h after wood smoke exposure but showed unexpected reductions in leukocyte numbers. The present study was performed to investigate responses at an earlier phase, regarding potential development of acute inflammation, as well as indications of cytotoxicity.

**Methods:**

In a double-blind, randomised crossover study, 14 healthy participants were exposed for 2 h to filtered air and diluted wood smoke from incomplete wood log combustion in a common wood stove with a mean particulate matter concentration of 409 µg/m^3^. Bronchoscopy with BW and BAL was performed 6 h after exposure. Differential cell counts, assessment of DNA-damage and ex vivo analysis of phagocytic function of phagocytosing BAL cells were performed. Wood smoke particles were also collected for in vitro toxicological analyses using bronchial epithelial cells (BEAS-2B) and alveolar type II-like cells (A549).

**Results:**

Exposure to wood smoke increased BAL lactate dehydrogenase (LDH) (*p* = 0.04) and reduced the ex vivo alveolar macrophage phagocytic capacity (*p* = 0.03) and viability (*p* = 0.02) vs. filtered air. BAL eosinophil numbers were increased after wood smoke (*p* = 0.02), while other cell types were unaffected in BW and BAL. In vitro exposure to wood smoke particles confirmed increased DNA-damage, decreased metabolic activity and cell cycle disturbances.

**Conclusions:**

Exposure to wood smoke from incomplete combustion did not induce any acute airway inflammatory cell influx at 6 h, apart from eosinophils. However, there were indications of a cytotoxic reaction with increased LDH, reduced cell viability and impaired alveolar macrophage phagocytic capacity. These findings are in accordance with earlier bronchoscopy findings at 24 h and may provide evidence for the increased susceptibility to infections by biomass smoke exposure, reported in population-based studies.

**Supplementary Information:**

The online version contains supplementary material available at 10.1186/s12989-023-00541-x.

## Background

Air pollution is a major global environmental cause of disease and premature death, contributing to over 6 million premature deaths annually, when taking both ambient and household air pollution into account [1]. Exposures to air pollution from biomass arise from multiple sources, both through ambient particles from residential biomass burning for heating and uncontrolled wild fires, as well as through indoor exposures, due to cooking in low- and middle-income countries. Globally, 2.4 billion people are dependent on biomass fuels for their everyday life [1]. Biomass burning is estimated to contribute to approximately 10–30% of the ambient fine particulate matter (PM) concentrations in different parts of Europe, depending on season and location [2]. Higher levels have been shown e.g. in northern Sweden, where small-scale residential wood combustion may occasionally contribute to up to 80% of the ambient PM_1_ (particulate matter with an aerodynamic diameter < 1 µm) [3]. Biomass combustion emissions are predicted to increase due to European environmental policies as well as a shortage of heating alternatives in the winter season. This has stimulated the use of renewable energy sources, including wood and other biomass fuels [4, 5]. With this comes a growing concern about health effects from exposure to biomass combustion emissions such as woodsmoke.

During biomass combustion in residential woodstoves and fireplaces, a wide range of gaseous and particulate components are formed and emitted with the flue gases, as cleaning devices are rarely installed or available. Regarding health effects, most attention has been given to exposure to PM, both in ambient and indoor environments [6, 7]. Particles are heterogenous and their physicochemical properties are heavily dependent on combustion conditions. Particles from wood smoke consist mainly of three classes; inorganic ash particles, soot agglomerates and organic-dominated particles [2, 8]. Stove technology as well as practical burning techniques are often not optimal, which may result in e.g. air-starved, incomplete combustion conditions leading to increased soot particle and polycyclic aromatic hydrocarbon (PAH) emissions [8].

In epidemiological studies, indoor exposure to biomass smoke has been associated with the development and worsening of chronic obstructive pulmonary disease (COPD), asthma and airway infections, including pneumonia and tuberculosis [9–12]. Hence, there is epidemiological evidence that exposure to biomass combustion emissions, especially from traditional wood burning, has a negative impact on human health, even if the mechanisms behind this response are still unclear. It has been proposed that PM from air pollution induces oxidative stress and inflammation in the lungs [9, 13, 14]. Alveolar macrophages are central in this response, as they phagocytose PM, release cytokines to recruit other inflammatory cells, and have a key role in clearance of bacteria and other pathogens [15, 16]. When it comes to traffic-related air pollution, e.g. diesel exhaust PM, inflammatory airway responses have been confirmed in a series of controlled short-term human exposure studies [17–21], but this has not been as evident for wood smoke.

Controlled human exposure studies give a unique opportunity to directly examine specific exposure responses to well-characterized air pollutants, however only a limited number of controlled human exposure studies addressing the health effects of wood smoke have been reported. So far the findings have been inconsistent with no, or only minor pro-inflammatory airway or systemic responses [21]. Sehlstedt et al. reported no increase in airway leukocytes in bronchial wash (BW) or bronchoalveolar lavage (BAL), but increased glutathione levels in BAL 24 h after exposure to smoke from a wood pellet boiler. This was suggested as a protective adaptive reaction against oxidative stress, limiting the inflammatory response [22]. Barregård et al. [23] reported signs of increased oxidative stress 20 h after wood smoke exposure, with increased malondialdehyde levels in breath condensate and elevated club cell protein in serum. Stockfelt et al. [24] reported an increase in club cell protein in the blood, 7 h post-exposure and a decrease in surfactant protein D post-exposure. Forchhammer et al. [25] examined three different PM concentrations in atopic subjects and reported no significant changes in any endpoint after wood smoke exposure including DNA damage, cell adhesion or cytokines in peripheral blood. Ghio et al. [26] reported an increase in the percent of neutrophils at 20 h after exposure to smoldering oak wood. A recent review of controlled human exposure studies highlighted wood smoke emission scenarios to vary between studies, and that complementary studies are warranted to improve insight into specific health effects and pathological mechanisms [21].

In a previous exposure study with wood smoke exposure from incomplete combustion in a wood stove in healthy subjects, we found an unexpected loss of macrophages, lymphocytes and neutrophils in BW at 24 h [27]. It was hypothesised that wood smoke would cause a neutrophilic inflammation, a classic hallmark of air pollution exposure, but the loss of cells at 24 h rather suggested a possible cytotoxic response. To verify these findings, and to exclude a transient leukocyte airway influx at an earlier time point, the current study was designed according to the same exposure set-up and principles as the preceding study, in order to evaluate early airway responses at 6 h post-exposure [27]. We hypothesised that wood smoke would cause an early acute airway neutrophilic inflammation together with indications of cytotoxicity.

## Results

### Exposure characteristics

Wood smoke was generated by burning birch wood logs in a commonly used Swedish wood stove for residential heating. The aim of the exposure conditions was to mimic the previous study by Muala et al. [27]. Thus, a common incomplete combustion operation with high burn rates and occasionally air-starved conditions was applied. The mean concentration of PM_1_ in the chamber was 409 ± 43 μg/m^3^ (filter based) during the exposures, with relatively high levels of soot and unburned hydrocarbons such as PAH. Exposure characteristics were quantified during all exposures, apart from PAH-PM, which was assessed from 10 exposures (Table 1).

**Table 1:** Exposure characteristics from the chamber compared with earlier performed study by Muala et al. [27]

		Present study	Muala et al. [27]
Exposure time		2 h	3 h
	Unit	Mean ± SD	Mean ± SD
PM_1_ mass concentration (TEOM)	μg/m^3^	453 ± 13	314 ± 38
PM_1_ mass concentration (filter)	μg/m^3^	409 ± 43	294 ± 36
CO	ppm	11 ± 2	25 ± 6
NO	ppm	0.21 ± 0.09	Not measured
NO_2_	ppm	0.02 ± 0.01	Not measured
NO_x_	ppm	0.23 ± 0.10	0.41 ± 0.12
EC/TC (elemental/total carbon)	ratio	0.78 ± 0.07	0.72 ± 0.08
Organic fraction of total PM_1_	%	27 ± 8	24 ± 8
Soot fraction of total PM_1_	%	62 ± 8	38 ± 9.9
Inorganic fraction of total PM_1_	%	10 ± 3	Not measured

Presented as mean values ± standard deviation (SD)

### Bronchial wash and bronchoalveolar lavage

**Fig. 1:**
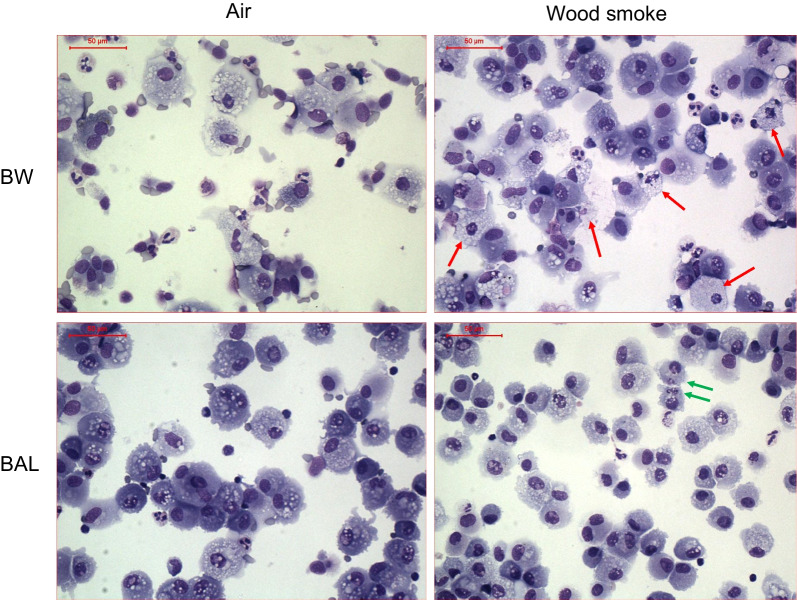
Images from cells from BAL and BW post air and post wood smoke exposure. Images were taken from cytocentrifuge preparations for cell differential counts and stained with May-Grünwald Giemsa. Upper left image: cells from BW post air exposure, upper right: cells from BW post wood smoke exposure, lower left: cells from BAL post air exposure and lower right: cells from BAL post wood smoke exposure. The red arrows indicate cells with nuclear condensation and fragmentation, typical for apoptosis induction. The green arrows indicate phagocytosing macrophages with engulfed neutrophils

Leukocyte differential counts were analysed in both BW and BAL representing proximal and distal airways, respectively, with no increase in neutrophil or lymphocyte numbers after wood smoke compared to filtered air exposure. However, a moderate but significant increase in eosinophils (*p* = 0.02) was found in BAL after wood smoke exposure, as shown in Table 2. In addition, a non-significant tendency to a reduction in the number of alveolar macrophages was found in BW after wood smoke exposure (*p* = 0.09). During the cell differential count the cells from bronchial wash post wood smoke exposure displayed signs of nuclear condensation and fragmentation, which is typical for apoptosis induction, see Fig. 1, upper right image.

**Table 2:** Cell counts in BW and BAL after exposure to wood smoke

	BW		*p*-value	BAL		*p*-value
Cells 10^4^/ml	Air	Wood smoke	Air vs. Wood smoke	Air	Wood smoke	Air vs. Wood smoke
Alveolar macrophages	7.9	5.1	0.08	9.9	9.9	0.90
	4.8–10.3	3.5–7.5		8.0–12.4	8.3–11.5	
Neutrophils	2.1	2.0	0.16	0.14	0.15	0.11
	1.3–2.6	0.93–3.9		0.06–0.34	0.07–0.66	
Lymphocytes	0.21	0.29	0.22	1.3	1.2	0.93
	0.12–0.47	0.01–0.63		0.82–2.0	0.70–2.5	
Eosinophils	0.00	0.01	0.11	0.00	0.03	**0.02**
	0.00–0.02	0.00–0.03		0.00–0.04	0.02–0.12	
Mast cells	0.003	0.006	0.08	0.007	0.008	0.55
	0.000–0.008	0.003–0.02		0.002–0.02	0.002–0.02	

Cell counts are given as cells × 10^4^/mL. Data are expressed as medians with interquartile range. P-values were calculated using the Wilcoxon signed-rank test. A p-value < 0.05 was considered significant and marked in bold

**Fig. 2:**
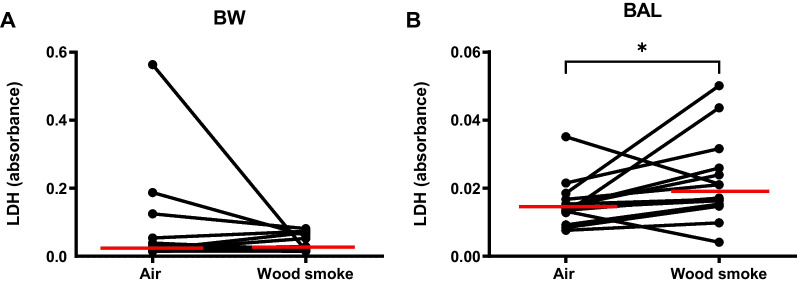
LDH measured in **A**: BW and **B**: BAL following exposure to wood smoke and filtered air. A statistically significant increase (p = 0.04) in BAL-LDH was found after wood smoke exposure. The red lines indicate median values. *Indicates p < 0.05, calculated using the Wilcoxon signed-rank test

Wood smoke exposure caused a significant increase in lactate dehydrogenase (LDH) in BAL (*p* = 0.04), but not in BW (Fig. 2).

**Fig. 3:**
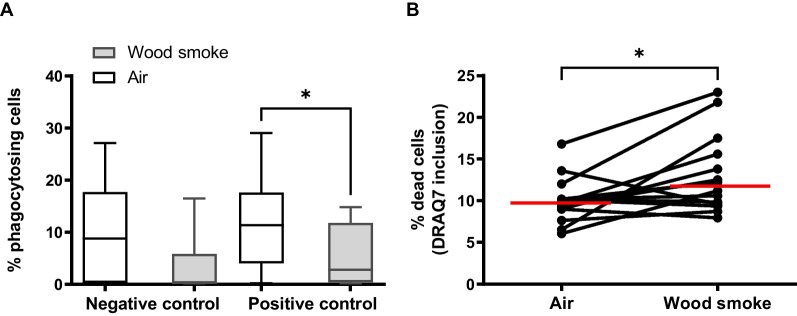
Phagocytosing cells in BAL. **A**: Phagocytosing ability measured in BAL cells after air and wood smoke exposures. Two different treatments; negative control (cells put on ice), positive control cells (incubated in 37 degrees Celsius). Whiskers indicate minimum to maximum value. **B**: Viability in BAL cells measured after phagocytosis evaluation. A statistically significant increase in dead cells was found (p = 0.02) among BAL cells exposed wood smoke. Red lines indicate median values and * indicates p < 0.05, calculated using the Wilcoxon signed-rank test

Phagocytosing cells in BAL displayed a decreased capacity to phagocytose bacteria after wood smoke exposure (*p* = 0.03). The viability of BAL cells after phagocytosis assessment was also reduced after wood smoke, measured by DRAQ7™ inclusion (*p* = 0.02), as shown in Fig. 3.

To evaluate genotoxicity in BAL and BW cells, DNA damage was assessed using the Comet assay. DNA damage was expressed as Olive tail moment (OTM). There were no significant differences detected between the two exposures (data in supplement, Additional files 1, 2).

### In vitro toxicity

**Fig. 4:**
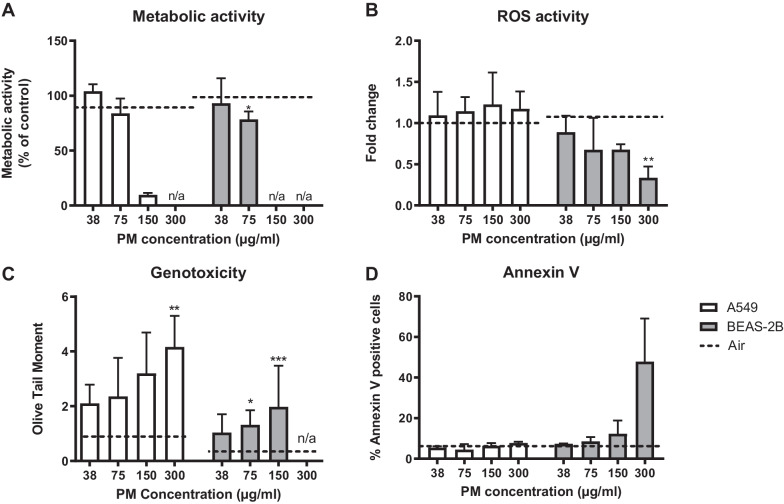
In vitro data showing; **A:** metabolic activity, **B**: ROS activity, **C**: genotoxicity and **D**: Annexin V, a marker of apoptosis. Two cell lines; A549 and BEAS-2B exposed to WSP at four different concentrations (38, 75, 150, 300 µg/mL) for 6 h. Controls were filtered air, blank and DMSO. Data are shown as median with IQR. Some concentrations could not be analysed due to extensive cell death, n.a. indicates not applicable. *indicates p < 0.05, **p < 0.01, and ***p < 0.001

Two cell lines; the alveolar type II-like cell line (A549) and the bronchial epithelial cell line (BEAS-2B) were exposed to filter-collected and extracted wood smoke particles (WSP) at four different concentrations. A significant decrease in metabolic activity, reflected by MTT-assay, was found in BEAS-2B cells at the 75 µg/mL WSP concentration (Fig. 4A). The cells at the 150 and 300 µg/mL concentrations could not be analysed in BEAS-2B, due to excessive cell death. Cellular reactive oxygen species (ROS) activity was measured using the 2′,7′-dichlorofluorescein (DCF) assay to assess oxidative stress in cells. A significant reduction in DCF fold change was found in BEAS-2B at a concentration of 300 µg/mL of wood smoke PM, with no significant changes in A549 cells (Fig. 4B).

DNA damage was assessed with Comet assay and expressed as OTM (Fig. 4C). A significant increase in DNA damage was found in A549 cells when comparing the highest concentration of WSP, 300 µg/mL, to air, whereas DNA-damage could not be analysed with the Comet assay at this concentration in BEAS-2B cells due to extensive cell death. Statistically significant increases in OTM were however observed in BEAS-2B cells exposed to 75 and 150 µg/mL WSP. No statistically significant difference was found between the control treatments (air, blank and dimethyl sulfoxide (DMSO)).

Apoptotic cell death was measured through quantification of Annexin V binding to externalised phosphatidylserine. Although there was an increase in Annexin V positive BEAS-2B cells at the highest dose of WSP, this was not statistically significant (*p* = 0.10) (Fig. 4D). There was also no increase in the percentage of apoptotic A549 cells after WSP exposure.

**Fig. 5:**
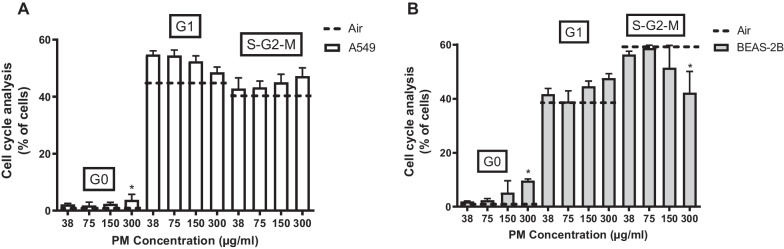
In vitro cell cycle analysis in two different cell lines; **A**: A549 and **B**: BEAS-2B exposed to WSP at four different concentrations (38, 75, 150, 300 µg/mL) for 6 h. Data are shown as medians with interquartile ranges and *indicates p < 0.05

Cell cycle phase distribution was assessed in cells after exposure to WSP. In A549 cells, a significant increase in cells in G0 phase was found at the highest concentration, 300 µg/mL. In BEAS-2B cells, a significant increase in cells in the G0 phase was also seen, as well as a decrease in cells in S-G2-M phase at 300 µg/mL (Fig. 5).

## Discussion

Contrary to our hypothesis, we could not detect any acute airway neutrophilia following exposure to wood smoke. In contrast, there were signs of cytotoxicity in BAL fluid, in terms of increased LDH levels, decreased cellular viability and reduced bacterial phagocytic capacity ex vivo. Furthermore, in vitro cell studies with wood smoke particles collected from the exposure chamber, showed decreased metabolic activity, increased DNA damage and cell cycle disturbances. We did not detect any signs of neutrophilic airway inflammation after wood smoke exposure, neither at 6 h nor at 24 h, which differs to findings from a series of controlled diesel exhaust and ozone studies [17, 18, 28].

A previous wood smoke exposure study in humans has reported an increase in the percentage of neutrophils in BAL and in blood 20 h after exposure to smouldering hardwood [26], but it is not clear whether the total number of neutrophils was increased. Rather than strong pro-inflammatory airway effects, data from previous wood smoke exposure studies in humans [22, 27] indicate possible cytotoxic effects and impairment of the immune defence. However, to be able to reject the hypothesis that wood smoke induces airway inflammation, responses in the airway epithelium and submucosa also need to be explored.

In BAL, a small, but significant increase in LDH was seen, indicative of cytotoxicity, as LDH is considered a marker for cell or tissue damage. LDH is an intracellular protein that is released upon damage of the cell membrane, which can be due to apoptosis, necrosis or other forms of cellular injury [29]. The source could be both inflammatory cell populations present in the airway lumen, and the bronchoalveolar epithelial surfaces and submucosa. BAL cells did show an impaired phagocytic capacity of bacteria ex vivo and also a reduced viability, reflected by flow cytometry techniques. The fact that the BAL alveolar macrophage cell numbers were not reduced, despite significantly impaired viability, could have been due to an influx of macrophages, in demand for increased clearance of combustion particles [30]. Such an event could have balanced a parallel loss of macrophages, as a result of impaired cell membrane integrity, as reflected by the increased LDH levels.

The tendency of a decrease in macrophages in BW is suggestive of an early sign of cytotoxicity at the 6-h time point in the proximal airways. This could have developed to a more established state of cytotoxicity at 24 h, as was present and demonstrated in terms of significantly reduced BW macrophages at the later time point in our preceding study [27].

Alveolar macrophages have a crucial role in the innate immune response. Macrophages are present in the respiratory lining fluid and serve as first line defence, as they phagocytose and kill airway pathogens intracellularly. One of their functions is also to recruit inflammatory cells to the lung and an impaired macrophage response can therefore affect the pulmonary inflammatory cascade negatively [14]. Here, we report decreased phagocytic capacity after wood smoke exposure. To our knowledge, this is the first report of decreased phagocytosing capacity by alveolar macrophages from human subjects already 6 h after exposure to wood smoke. The phagocytosis analysis utilised in this manuscript uses macrophages from the bronchoalveolar compartment obtained through BAL, which were exposed in a realistic scenario with high internal validity. Our finding here with decreased phagocytic function may provide a possible explanation to the increased incidence of respiratory infections associated with wood smoke exposure [10, 31]. There has been reports of affected phagocytosis among BAL cells in response to other air pollutants, such as diesel exhaust [30]. Decreased phagocytic function has also been shown in macrophages obtained through BAL from humans chronically exposed to household air pollution [32], and decreased ability to restrict growth of *Mycobacterium tuberculosis* has been reported in monocyte-derived macrophages exposed in vitro to PAHs derived from wood smoke, despite a dose-dependent increase of pro-inflammatory activation markers among the macrophages [33]. Decreased phagocytic activity and higher bacterial load have also been reported in COPD patients chronically exposed to biomass smoke compared to tobacco smoke-associated COPD [34]. That study used monocyte-derived macrophages extracted from peripheral blood and assessed phagocytic function comparing the different COPD groups with normal subjects, which suggests that biomass smoke may affect monocyte and macrophage phagocytosis more than tobacco smoke.

A small, but statistically significant, increase in lavage eosinophils was found after wood smoke exposure. Eosinophils are known to have a key role in asthma and higher levels predict worsening of the disease. In the present study, none of the participants were allergic or had asthma but nevertheless responded with an increase in airway eosinophils. Air pollution has been considered to contribute to the development and worsening of asthma due to oxidative responses and immune dysregulation [35, 36]. Interestingly, the present findings are in accordance with our previous observations of increased eosinophils in BAL in healthy non-allergic, non-atopic participants at 6 h after exposure to diesel exposure [20]. Several studies have highlighted the eosinophilic response to carbon nanotubes, indicated as noxious man-made industrial products [37–39]. Further research investigating human bronchial mucosal biopsies may increase the understanding of the interaction between air pollution on eosinophilic airway inflammation and airway disease.

In the present study, the mean PM_1_ concentration in the chamber was 409 µg/m^3^. Thus, the particle concentration in the chamber was high, but still reflects real-life exposure scenarios with indoor biomass burning [40, 41]. The wood smoke contribution to PM levels in Europe, North America, Australia and New Zealand has been shown to typically correspond to season-based averages of 10–30 µg/m^3^, with outdoor peak concentrations of 100 µg/m^3^ or more [2]. Earlier controlled human exposure studies to wood smoke have been performed with PM concentrations ranging from 180 to 1115 µg/m^3^, but since exposure time has varied (1–4 h), the comparable exposure doses (time x concentration) fall between 380 and 1115 µg [21]. Since we aimed at comparing the results of the present study with earlier findings [27], an exposure dose close to our previous and most recent study was chosen. The exposure time was shortened from 3 to 2 h and the concentration increased correspondingly, in order to perform exposure and bronchoscopy on the same day, at a six-hour interval.

In this study wood smoke from incomplete combustion conditions was used, resulting in PAH and soot rich PM, compared to more efficient biomass burning applications. The physicochemical properties of the particles are crucial for their toxicological properties and associated health effects [8]. WSP tend to be more heterogenous than for example diesel exhaust particles, as combustion conditions might vary considerably and affect the physicochemical properties. Compared to our preceding study [27], the exposure characteristics were relatively similar except for the PAH-PM associated levels, which were notably elevated. PAHs are formed during incomplete combustion of biomass, as a product of thermal conversion of primary and secondary pyrolysis products, at high temperatures without access of oxygen [42]. The formation and degradation (oxidation) are heavily sensitive to specific combustion conditions, where short events can contribute to very high emission levels. In this work, we have no clear explanation for the higher PAH level compared to the previous study, since all other available data indicate that the combustion conditions in the stove and exposure conditions in the chamber were very similar. Still, PAH analysis results may differ from different exposure occasions and, since different analysis methods for determining the PAHs were employed for the two studies, this can be part of the explanation for divergent PAH data.

In vitro exposure to WSP at concentrations above 75 µg/mL resulted in a concentration-dependent increase in DNA damage compared to air exposed control cells, but no increase in DNA damage was seen in BW and BAL after wood smoke exposure. DNA damage was expected, due to the high levels of PAH during the exposures, as many PAH compounds cause oxidative DNA damage and are well-known carcinogens [43, 44]. Lack of DNA damage in short-term exposure scenarios in humans has previously been reported in peripheral blood mononuclear cells (PBMC) [25, 45]. However, an increase in DNA-repairing gene expression in PBMC after wood smoke exposure suggests that DNA repair function may be enhanced in response to short-term wood smoke [45]. In chronic exposure scenarios DNA damage has been reported, indicating that the DNA repairing mechanisms may not be able to manage with the repeated insults [46, 47].

Human lung epithelial cell lines were exposed to WSP collected from the human chamber exposures. The cell lines utilised were chosen to investigate how airway epithelial and alveolar cells may be affected, as well as to investigate toxicological mechanisms. A549 cells are a cancer cell line derived from an adenocarcinoma and are commonly used as a model of type II pulmonary epithelial cells. Overall, the BEAS-2B cells showed a higher sensitivity to WSP than A549 cells, with extensive cell death at concentrations of 150 and 300 µg/mL. This may be due to the different origins of the cell lines, with A549 originating from a pulmonary adenocarcinoma and therefore may be more resistant to the effects of WSP, whereas the BEAS-B2 cells are from an immortalized cell line derived from a normal bronchial epithelium. [48, 49]. This difference may also be reflected in the higher apoptosis rate among the BEAS-2B cell line compared with A549.

The standard exposure procedure in air pollution experiments in vitro has been to resuspend PM in liquid growth media. This may alter the particle composition, since adding e.g. nano particles directly in culture medium increases the possibility of particle agglomeration, which can affect the particle toxicity [50]. In vivo cells are protected via the respiratory tract lining fluid and immune cells present within the airway epithelium, a scenario that is difficult to mimic in an in vitro setup. The method still remains valuable when elucidating the cellular mechanisms behind cell toxicity, while recognizing that complementary techniques including primary cells, 3D cultures and air liquid interface have potential for enhanced precision, in complementary studies [51]. The doses chosen in the in vitro exposures was based on preceding experiments designed to identify cellular effects [52]. It is recognized that in vitro studies often demands considerably higher concentrations than encountered locally in the human lung after inhalation of woodsmoke [53].

We also found an unexpected reduction in cellular ROS activity in BEAS-2B cells, since WSP have previously been reported to cause an increase in intracellular ROS activity [54]. ROS activity was measured through fluorescence using the DCF assay, and the decreased fluorescence is expected to be related to the increased cell death and, consequently, reduced production of ROS.

### Strengths

With this study we can provide detailed data on the local airway response following a short-term exposure to a soot and PAH-rich wood smoke. The exposure set-up and combustion situation are well validated and controlled by combustion aerosol scientists, also responsible for the physiochemical characterisation of the wood smoke. We used bronchoscopy to perform local sampling from both the proximal and distal airways, as reflected by BW and BAL, to highlight the responses in different regions of the lungs. The blinded randomised cross-over design provides high internal validity. Collected wood smoke particles from the experiments were further investigated by in vitro systems, reflecting bronchial epithelium and the alveolar region, in order to gain complementary information.

### Limitations

Controlled exposure studies are usually limited to a single exposure and give information of a certain exposure level and time point, contrary to animal and cell experiments, which allows for larger numbers of scenarios to be investigated. In this study we failed to reach sex balance. Although both male and female subjects were included in the study, we were unable to perform any gender analysis in response to wood smoke, due to the low number of included female subjects. Further studies are warranted to assess if there are gender differences in response to wood smoke, especially when women are predominantly exposed in low- and middle-income countries. An earlier time point for bronchoscopy was chosen in the present study to investigate early airway responses to wood smoke. While aiming for a similar exposure dose (time x exposure concentration) as in the previous study at 24 h post exposure, a shorter exposure time and hence a higher concentration was required to manage logistics for the research subjects, medical staff and laboratory labour. As earlier alluded the PM concentrations in the in vitro experiments were considerably higher than encountered locally in the human lung in vivo*.*

## Conclusions

Exposure to wood smoke from incomplete combustion did not induce any acute inflammatory cell influx detectable in BW or BAL at 6 h, apart from eosinophils. Instead, there were signs of early cytotoxic reactions with increased LDH, reduced cell viability and impaired alveolar macrophage phagocytic capacity. These findings are in accordance with the preceding bronchoscopy study with sampling at 24 h, as well as in vitro cell data. Our findings provide some evidence for the increased susceptibility to infections and worsening of respiratory diseases by biomass smoke exposure, as reported in population-based studies [10–12, 31]. Further characterization of the responses in the human airway submucosa and peripheral blood is needed to provide a clearer picture of the potentially negative health effects of wood smoke.

## Methods

### Participants

Fourteen healthy, non-smoking individuals with normal lung function (mean age 26, range 19–35 years, 3 females, 11 males) were recruited. All participants gave their written informed consent. A physical examination, dynamic spirometry (FEV_1_, FVC and FEV_1_/FVC), 12 lead electrocardiogram, baseline blood count and renal function assessment were performed prior to inclusion. None of the participants had a history of atopy or allergy. Exclusion criteria were presence of diabetes, cardiovascular disease, renal or hepatic failure, systolic blood pressure below 100 mmHg, blood donation three months before study start, and earlier or ongoing occupational air pollution exposure. All participants were free from airway infection 6 weeks prior to exposure and no anti-inflammatory or vitamin supplements were permitted for the duration of the study. The study was approved by the Regional Ethical Review Board (EPN) in Umeå (Diary number: 2017-06-13, 2017/250-31) and performed according to the declaration of Helsinki [55].

### Study design and exposure procedures

The study was performed employing a double-blinded, randomised cross-over design, with each participant undergoing two controlled exposures, once to filtered air and once to wood smoke, with at least 3 weeks apart. Exposures lasted 2 h and were carried out in a stainless-steel exposure chamber (15.3 m^3^) at the Thermochemical Energy Conversion Laboratory at Umeå University. Participants alternated between rest and exercise on a bicycle ergometer at 15-min intervals to achieve a minute ventilation of 20L/min/m^2^ body surface. Perceived intensity of symptoms was recorded before and during the exposures, every 30 min, according to the modified Borg scale [56].

Wood smoke was generated in a common residential Swedish wood stove, commonly installed during the 1990s, fired with birch wood logs (16–18% moisture content) under incomplete combustion conditions, targeting a PM_1_ concentration of 400 µg/m^3^ in the exposure chamber. The combustion procedures included a high frequency of fuel addition to achieve a high burn rate with partial air-starved conditions, well-known to cause elevated levels of products of incomplete combustion such as CO, PAH and soot [8, 57]. The combustion and exposure set-up and wood burning procedures, were in accordance with previous research [27, 58].

### Exposure characteristic and PM measurements

Combustion conditions were monitored by measuring O_2_ and CO in the flue gases. 2–15% for oxygen and 1000–7000 ppm for CO, with peaks at 18 000 ppm during the highest burn rates, shortly after fuel addition. To achieve the desired PM concentration, wood smoke was diluted in three steps with a high efficiency particulate air (HEPA) filter and an activated carbon filter before reaching the chamber. To ensure participant safety, NO_x_ and CO concentrations in the chamber were monitored continuously using chemiluminescence (CLD 700 Ecophysics, > 0.001 ppm) for NO_x_ and IR (UNOR6N Maihak) for CO. PM_1_ mass concentration was measured with a TEOM 1400 (Thermo Scientific). PM_1_ concentration in the chamber was 409 ± 43 (mean ± SD) µg/m^3^, filter based. A scanning mobility particle sizer (SMPS) was used to measure the particle number concentration and size distribution, based on mobility diameter, of the aerosol in the chamber. The fraction of filter-based organic carbon (OC) and elemental carbon (EC) of the PM in the chamber were determined by thermal-optical carbon analysis (Method NIOSH 5040), according to standard procedures [59]. To convert the OC content to total organic PM, a factor of 1.8 was used, and to convert EC to soot PM mass concentration, a factor of 1.1 was used [59].

Measurements of twelve different particulate bound pure PAHs in the chamber was performed during ten exposures through sampling with polytetrafluoroethylene (PTFE) coated glass fibre filters (Ø 47 mm). The filters were extracted using pressurized liquid extraction and purified on columns with KOH-impregnated silica gel eluted with dichloromethane, after which the eluate was evaporated, and the solvent exchanged to toluene. The samples were then analysed with gas chromatography (GC) high-resolution mass spectrometry (HRMS) operated in electron ionization mode. To identify target compounds, GC retention data for the samples were compared with reference standards. A lab blank was run, and the number of individual compounds in the blank was below 10% of the amount found in corresponding samples, thus no blank corrections were performed. Twelve different PAH compounds were quantified, and the total PAH concentration in the chamber was 6.46 ± 2.72 μg/m^3^. In our previous study the total PAH concentration was 0.78 ± 0.56 μg/m^3^ [27]. The five dominating PAH compounds were (in descending order); benzo(k)fluoranten, benzo(a)pyren, indeno(1,2,3-cd)pyren, benzo(g,h,i)perylene and chrysene, accounting for 68–87% (min–max) of the total analysed PAH.

### Bronchoscopy

Bronchoscopy was performed 6 h after exposure with a flexible video bronchoscope (EB-580S, FUJIFILM Corporation, Japan). Topical anaesthesia with lidocaine was applied to the pharynx and bronchial tree. Bronchial wash (BW) 2 × 20 ml and Bronchoalveolar lavage (BAL) 3 × 60 ml with sterile saline were performed. Aspirates from BW and BAL were placed on ice in separate siliconized containers. Lavage samples were filtered through a nylon filter (100 µm) and centrifuged at 400 × g for 15 min. Cell pellets were resuspended in 0.9% NaCl at a concentration of 10^6^ cells/mL. Differential cell counts were performed on cytocentrifuge preparations stained with May-Grünwald Giemsa. 400 cells per slide were counted. Images were taken with LeicaQWin V3 (Leica Q500IW; Leica, Cambridge, UK).

### LDH

Lactate dehydrogenase (LDH) was analysed to assess cell toxicity in supernatants from BAL fluids and BW using the LDH kit (Roche, Basel, Switzerland) according to the manufacturer’s instructions.

### Phagocytosis

Phagocytosing ability was measured in 1 × 10^5^ BAL cells. Cells were diluted in PBS containing 10% Fetal bovine serum (FBS). To assess phagocytic activity 20 pHrodo™ E. coli BioParticles per cell were added. Cells were incubated at 37 °C for 60 min (positive control) or incubated on ice for 60 min (negative control).

Samples were then put on ice and ice-cold PBS was added to stop reactions. Tubes were then centrifuged at 3,200 rpm and resuspended in PBS containing 2% FBS. 3µL DRAQ7™ dye (Abcam, Cambridge, UK) was added as a viability marker and dead cells were quantified and excluded through DRAQ7™ inclusion. The samples were analysed using the BD Accuri flow cytometer (BD Biosciences, Stockholm, Sweden), with pHrodo™ and DRAQ7™ excited with the 488 nm laser and 640 nm lasers then detected in the FL-2 and FL-4 channels, respectively. 20,000 gated events were analysed.

### Cell culture

All materials were purchased from Sigma (St Louis, Missouri, USA) unless otherwise stated. The alveolar type II-like cell line A549 was cultured in Dulbecco Modified Eagle Medium (DMEM) supplemented with 10% (v/v) heat-inactivated FBS, 2 mM L-glutamine and 100 U/mL penicillin/streptomycin. The bronchial epithelial cell line BEAS-2B was cultured in bronchial epithelial growth media (BEGM; Lonza) supplemented with BEGM bullet kit (Lonza) and grown on tissue culture plastic coated with 0.01 mg/mL fibronectin, 0.03 mg/mL collagen and 0.01 mg/mL bovine serum albumin (BSA). Cells were cultured in a humid atmosphere at 37 °C, 5% (v/v) CO_2_.

The day before exposure, A549 cells were seeded at a density of 1.2 × 10^5^ cells/well and BEAS-2B cells at 2.5 × 10^5^ cells/well in 12-well plates (VWR). Culture medium was changed 1 h before cells were exposed to particles, or their controls.

### PM sample preparation

Wood smoke particles were collected onto PTFE substrates from the chamber with a 5-stage (> 2.5 µm to < 0.2 µm) gravimetric impactor (DGI, Dekati Ltd., Finland). Particle collecting and blank control filters were eluted in high pressure liquid chromatography grade methanol under sonication, twice for 30 min at 22 °C (room temperature). PM suspensions were pooled, and excess methanol was evaporated using a rotary evaporator at 30 °C under vacuum. The concentrated PM sample was then aliquoted, calculated on a mass basis, dried in glass tubes under nitrogen flow and stored at -20 °C.

### In vitro exposure

PM samples were resuspended in DMSO (20 µL/mg PM) 30 min before exposure, sterile distilled water was added to make the stock concentration (5 mg/mL). Samples were then sonicated for 30 min at room temperature to obtain a homogenous mixture. Cell lines were exposed to WSP at four different concentrations: 38, 75, 150 or 300 µg/mL for 6 h at 37 °C, 5% CO_2_. After exposure, the supernatant was removed, aliquoted and frozen, and cells were detached using trypsin. Heat-inactivated FBS (10%) was added to block trypsin activity and cells were centrifuged at 900 x*g* at 4 °C for 5 min. Cell pellets were re-suspended in PBS containing 2% FBS for downstream analysis.

### Cell cycle analysis

Detached cells were fixed and permeabilized in 70% ethanol. Fixed cells were first treated with RNAse A (150 μg/mL) and then cellular DNA was stained using propidium iodide (8 μg/mL; Sigma, St Louis, MO, USA). Samples were analysed on the BD Accuri flow cytometer (BD Biosciences, Stockholm, Sweden) and 30,000 gated events (single cells) were collected for analysis using FlowJo V10 (FlowJo; LLC, Ashland, OR, USA), as previously described [48].

### Metabolic activity

The metabolic activity of BEAS-2B and A549 cells was analysed using the MTT test on 96-well plates. The absorbance was detected at 570 nm using a spectrophotometric plate reader and the proportion of metabolically active cells was calculated as a percentage from corresponding readings of the blank control cells. The absorbance of the PM was measured in blank samples at the different concentrations and adjusted for in the calculations.

### Apoptosis

Apoptosis was assessed using an Annexin V kit (Invitrogen, Eugene, USA). Manufacturer’s protocol was followed except for using 7-ADD instead of PI inclusion. Samples were then analysed using BD Accuri flow cytometer excited with 530 nm (FL3-7ADD and FL4-APC, Annexin V) and expressed as percent apoptotic cells of the cell population.

### Oxidative stress

Oxidative stress was assessed in vitro through 2′, 7′-dichlorodihydrofluorescein diacetate (DCF) assay, which measures intracellular ROS activity. Cell suspensions were analysed on a 96-well plate. DCF stock solution (5 mM DCF reagent (Sigma) dissolved in DMSO) was added diluted 1:10 with PBS. The plate was incubated for 30 min at 37 °C before fluorescence was measured (excitation 485 nm, emission 530 nm). Fold change of ROS activity was calculated using the blank control.

### Genotoxicity analysis

DNA damage was detected through alkaline single cell gel electrophoresis (Comet assay). Cell suspensions were mixed with 0.5% low-melting point agarose (VWR) and seeded onto glass slides coated with 1% normal agarose (VWR) in duplicate. Slides were incubated with lysis buffer (2.5 M NaCl, 100 mM Na_2_EDTA, 10 mM Tris, 1% Triton X-100, pH 10) for 1 h, then washed with neutralisation buffer (0.4 M Tris, pH 7.5). Slides were allowed to equilibrate in an alkaline electrophoresis buffer (1 mM EDTA, 300 mM NaOH, pH > 13, 40 min) before electrophoresis was performed for 20 min (constant 24 V /300 mA). Slides were then neutralised and dehydrated with 99% EtOH.

Cell nuclei were stained with ethidium bromide (20 µg/mL) and imaged using an Olympus BX51 microscope and Olympus DP71 camera under a 20 × objective. Comets were analysed using the free comet analysis software Casplab_1.2.3b2 (CaspLab.com). For each duplicate, 50 and 100 cells were analysed for in vitro, and lavage cells (BW and BAL), respectively. OTM [(tail mean–head mean) x tail% DNA/100)] was calculated and used in statistical analyses. All samples from each individual subject were analysed simultaneously to minimise inter-assay variation.

### Statistics

All data were normality tested with the Shapiro–Wilk test. Non-parametric statistical analyses were performed for comparison of BW and BAL data. Wilcoxon signed rank test was used for the paired BW and BAL. The measured responses in vitro of A549 and BEAS-2B were analysed with non-parametric statistical analyses with Kruskal–Wallis test, with Dunn’s multiple comparison test. Values of *p* < 0.05 were considered significant. Statistical analyses were performed with Prism 9 (Graph Pad software for Windows, San Diego, USA) and SPSS, version 26 for Windows (IBM® SPSS® Statistics 20, Chicago, USA).

## Supplementary Information

**Additional file 1. Supplement 1**. DNA-damage in BAL and BW. Graph of DNA-damage in BAL and BW after air and wood smoke exposure.

**Additional file 2. Supplement 2**. Comet assay from BW and BAL. Pictures of BAL and BW cells from the comet assay.

## Data Availability

All relevant data are included in the manuscript and supporting information. These are also available from the authors upon request.
