# Supplementary material for: Reduced bronchoalveolar macrophage phagocytosis and cytotoxic effects after controlled short-term exposure to wood smoke in healthy humans
Source: Part Fibre Toxicol. 2023 Jul 31;20:30. doi: 10.1186/s12989-023-00541-x (PMC10388518; doi:10.1186/s12989-023-00541-x)
Supplement: Supplementary file 1 — Additional file 1. Supplement 1. DNA-damage in BAL and BW. Graph of DNA-damage in BAL and BW after air and wood smoke exposure. [file 12989_2023_541_MOESM1_ESM.pdf]

Supplement 1. DNA-damage in BAL and BW

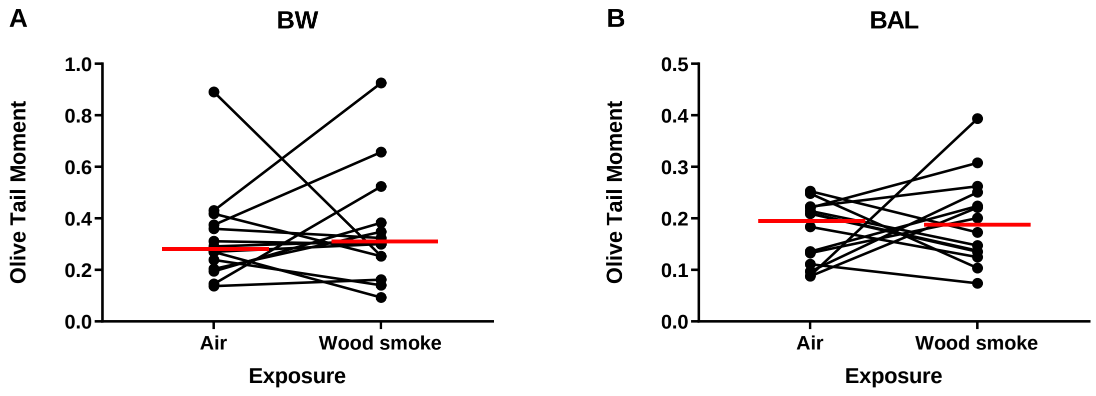

Supplement 1. *DNA damage measured in* ***A:*** *bronchial wash and* ***B:*** *bronchoalveolar lavage following exposure of healthy subjects to wood smoke or filtered air. DNA damage was quantified using the Comet assay and expressed as Olive tail moment. No statistically significant change was seen between the exposures. The red line indicates median value.*
