# Supplementary material for: Reduced bronchoalveolar macrophage phagocytosis and cytotoxic effects after controlled short-term exposure to wood smoke in healthy humans
Source: Part Fibre Toxicol. 2023 Jul 31;20:30. doi: 10.1186/s12989-023-00541-x (PMC10388518; doi:10.1186/s12989-023-00541-x)
Supplement: Supplementary file 2 — Additional file 2. Supplement 2. Comet assay from BW and BAL. Pictures of BAL and BW cells from the comet assay. [file 12989_2023_541_MOESM2_ESM.pdf]

Supplement 2. Comet assay from BW and BAL

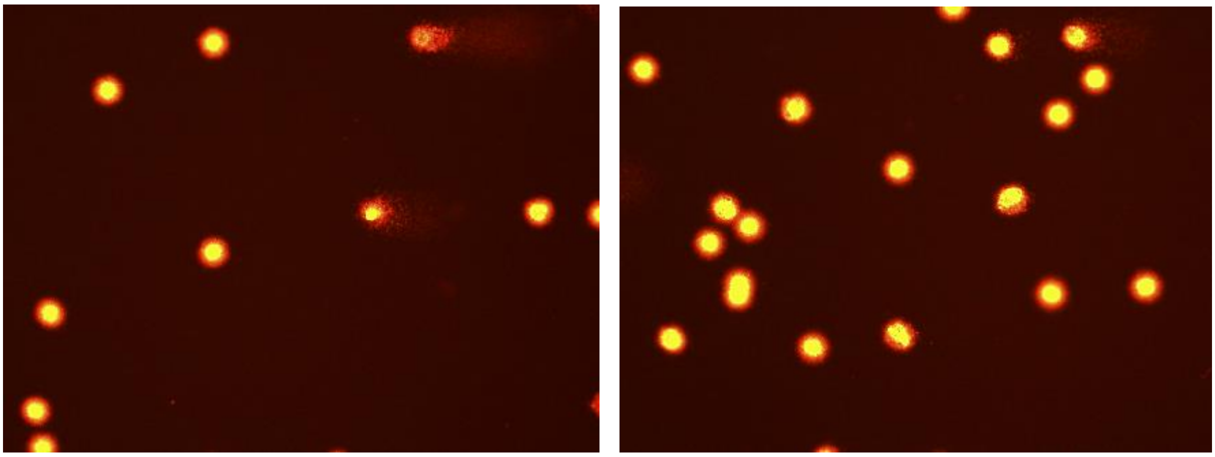

Supplement 2. *Comet assay performed on cells from bronchial wash (left) and bronchoalveolar lavage (right).*
